# Breast schwannoma in a patient with diffuse large B-cell lymphoma: a case report

**DOI:** 10.1186/1752-1947-6-423

**Published:** 2012-12-17

**Authors:** Ayse Salihoglu, Sinem Nihal Esatoglu, Ahmet Emre Eskazan, Metin Halac, Seniz Ongoren Aydin

**Affiliations:** 1 Istanbul University, Cerrahpasa Faculty of Medicine, Department of Internal Medicine, Division of Hematology, Kocamustafapasa, Fatih, Istanbul, Turkey; 2 Diyarbakir Training and Research Hospital, Department of Hematology, Diyarbakir, Turkey; 3 Istanbul University, Cerrahpasa Faculty of Medicine, Department of Nuclear Medicine, Kocamustafapasa, Fatih, Istanbul, Turkey

**Keywords:** Breast, Diffuse large B-cell lymphoma, Positron emission tomography-computed tomography, Schwannoma

## Abstract

**Introduction:**

Schwannomas are mostly benign tumors arising from Schwann cells of the nerve sheaths. Breast schwannomas are very rare and account for only 2.6% of cases. As far as we know this is the first reported case of breast schwannoma discovered in a patient with diffuse large B-cell lymphoma. The breast schwannoma was evaluated with positron emission tomography and it exhibited moderate 18F-fluorodeoxyglucose uptake.

**Case presentation:**

We present the case of a breast schwannoma in a 63-year-old Caucasian woman who was diagnosed with diffuse large B-cell lymphoma.

**Conclusion:**

Imaging modalities including positron emission tomography-computed tomography failed to distinguish breast schwannoma from diffuse large B-cell lymphoma involvement of the breast.

## Introduction

Schwannoma is a slow-growing tumor arising from the nerve sheath of peripheral, cranial and autonomic nerves. It commonly occurs in the neck, head and extensor surfaces of the extremities [1,2]. A schwannoma is usually solitary and presents several years before diagnosis [3]. Breast-located schwannomas are unusual. Das Gupta *et al*. reported the percentage of schwannomas arising in the breast to be only 2.6% of all schwannomas [4]. Diffuse large B-cell lymphoma (DLBCL) constitutes approximately 30% of all lymphomas and is the most common subtype throughout the world [5]. We report here a case of schwannoma arising in the breast of a 63-year-old Caucasian woman treated for DLBCL. To the best of our knowledge this is the first reported case of schwannoma co-existing with DLBCL and detected by positron emission tomography (PET).

## Case presentation

**Figure 1:**
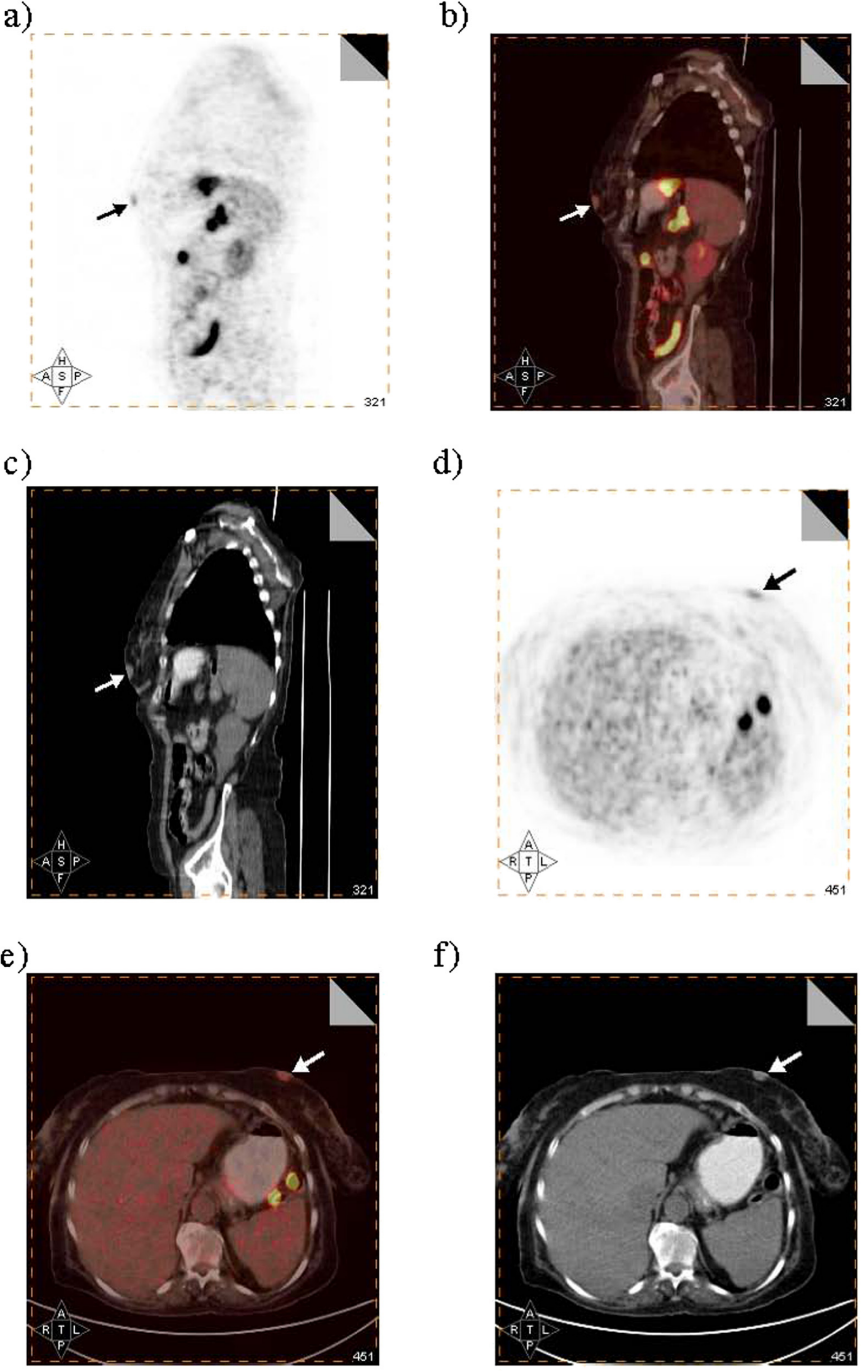
Sagittal (a, b, c) and axial (d, e, f) positron emission tomography-computed tomography images demonstrating a small focal region of moderate fluorodeoxyglucose uptake within the left breast (arrows) compared to normal parenchyma.

**Figure 2:**
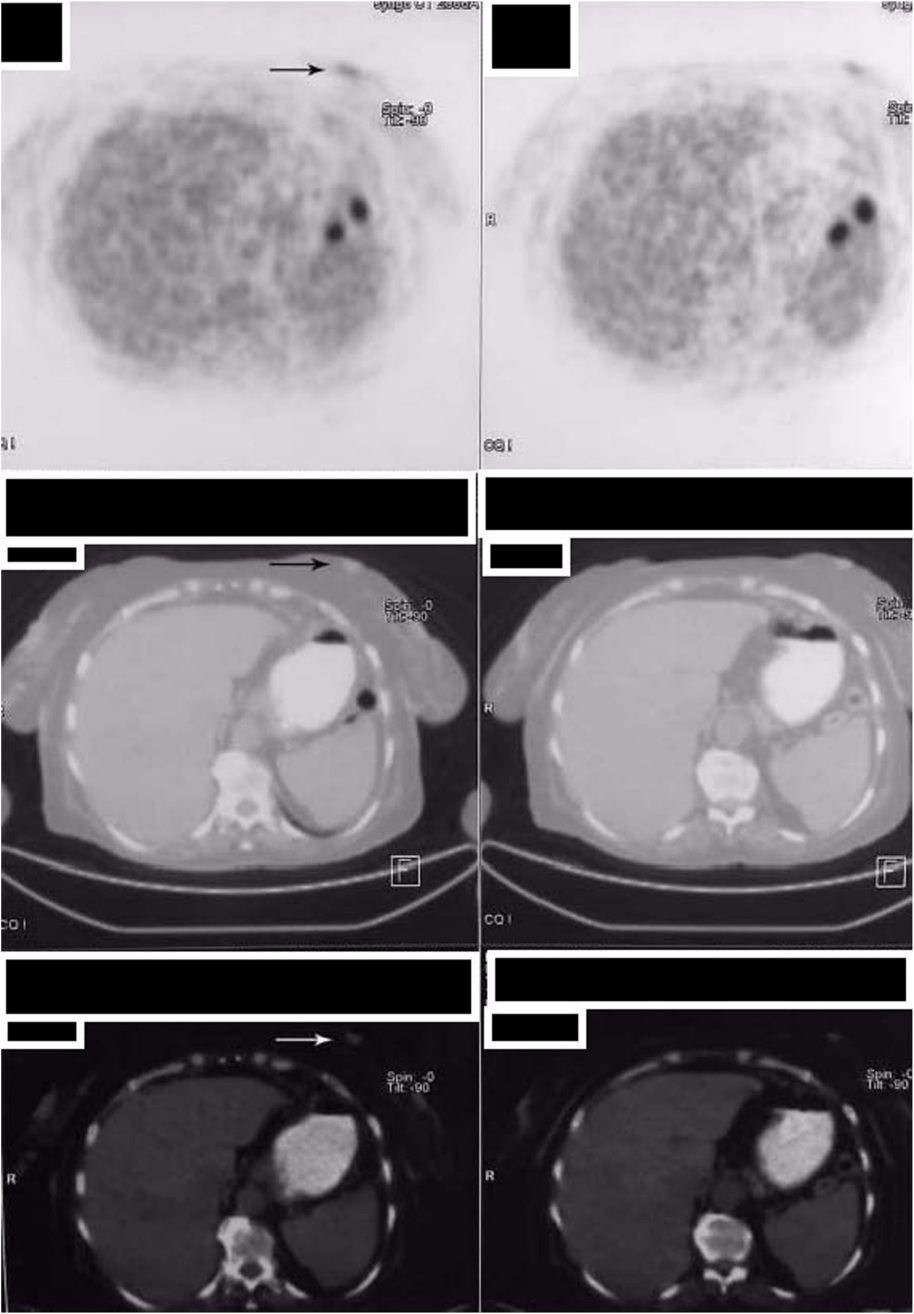
The positron emission tomography-computed tomography images showing hypermetabolic mass lesion in the lower inner quadrant of the left breast (selected arrows).

**Figure 3:**
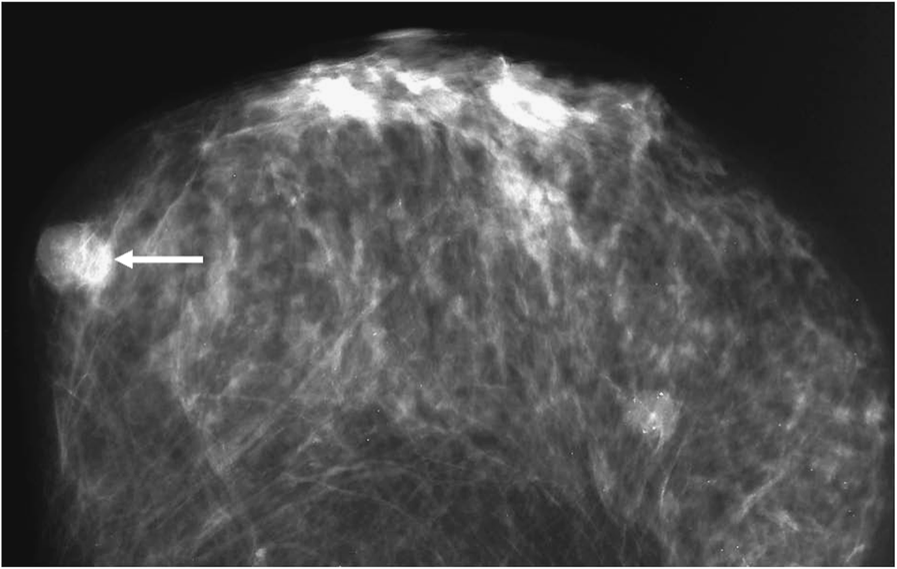
Craniocaudal projection of the mammography showing round circumscribed mass with sharply defined margin in the inner part of the breast (arrow).

**Figure 4:**
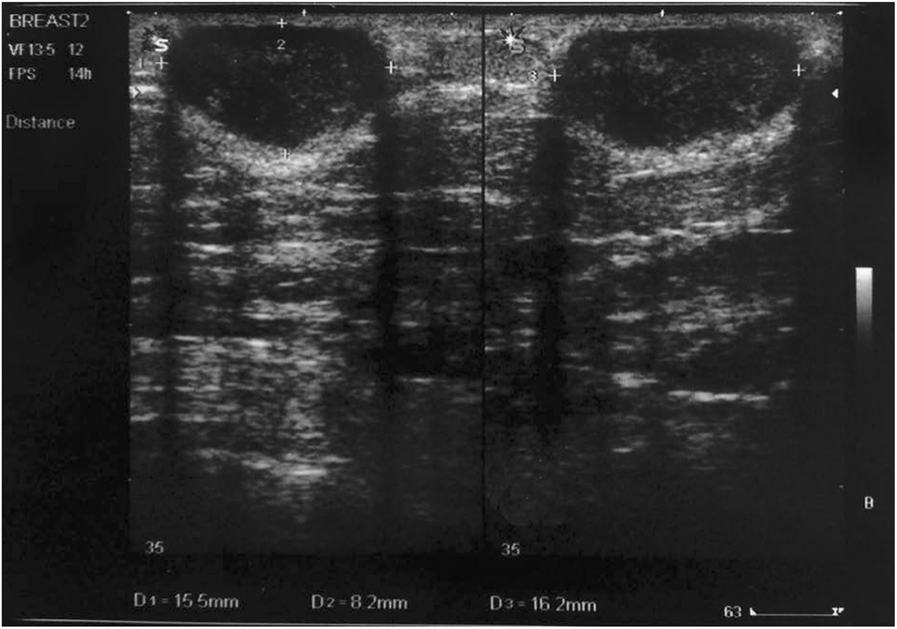
Ultrasonographic image demonstrating a well-defined hypoechoic ovoid mass with low amplitude internal echoes and reflective shadowing at the edges.

**Figure 5:**
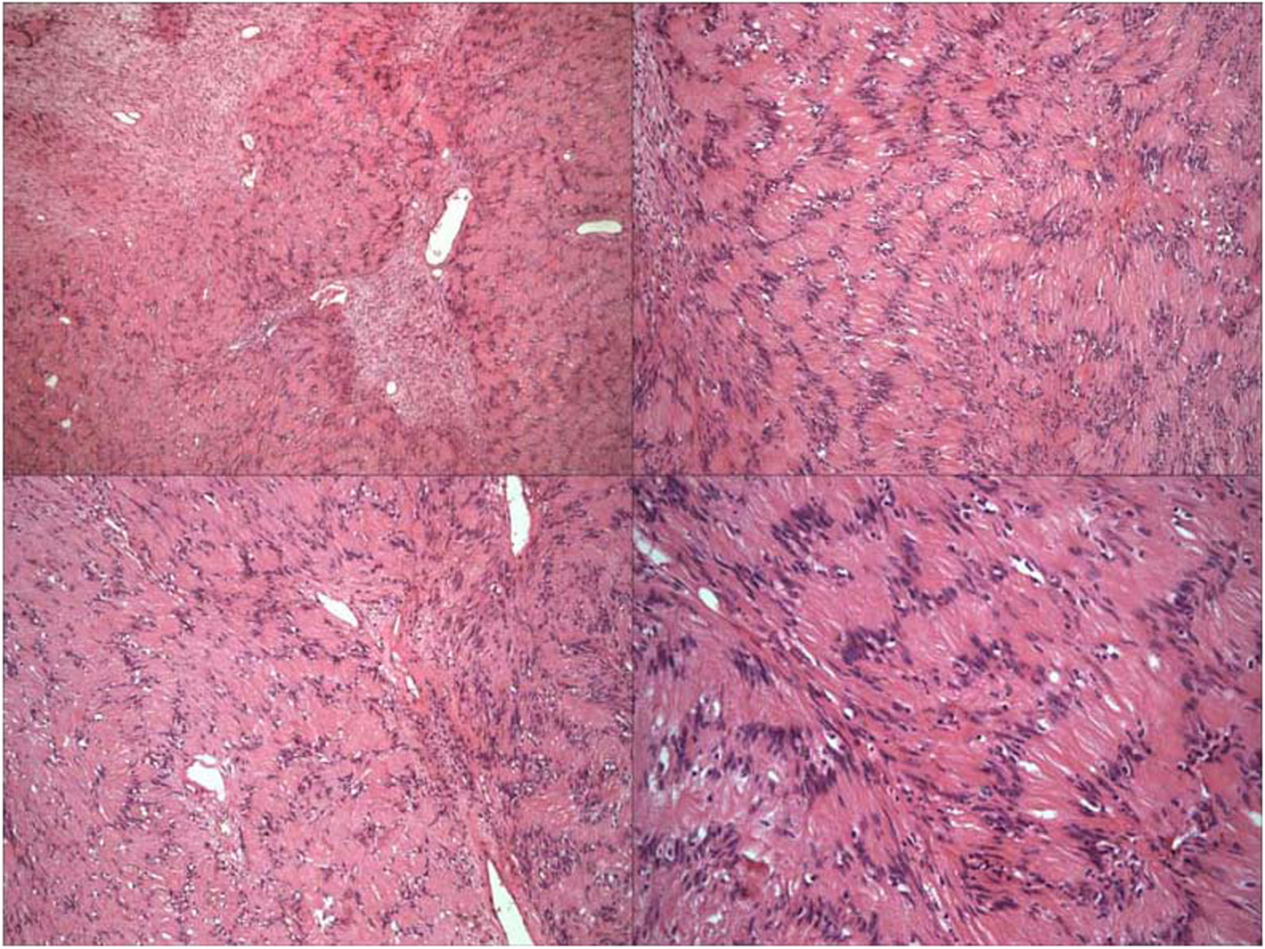
Spindle cells arranged in palisading fashion (hematoxylin and eosin × 40-100-100-200).

A 63-year-old Caucasian woman presented with a three-month history of pelvic pain, urinary and fecal incontinence and palpitations. Past medical and surgical histories were negative. On physical examination the patient appeared ill. A rapid heart rate was palpated which corresponded to atrial flutter on electrocardiography. Physical examination also revealed a left breast mass, which the patient reported to be there for 25 years. It was a nontender, mobile, elastic hard and well-circumscribed mass, two × one cm in size, with a smooth surface located in the left lower inner quadrant. Both axillae and supraclavicular fossae were negative on palpation. No signs of nipple discharge and skin changes were apparent. A thoracic computed tomography (CT) scan revealed a mediastinal mass causing external compression of the heart. The breast mass was identified on thoracic CT as a hypodense lesion of one cm in diameter. Magnetic resonance imaging (MRI) of the abdomen and pelvis exhibited multiple lymphadenopathies. A diagnosis of non-Hodgkin’s lymphoma of diffuse large B-cell type was established by Tru-Cut® needle biopsy of the pelvic lymphadenopathy. The patient’s general condition necessitated immediate initiation of chemotherapy without further evaluation of the breast mass. A PET scan scheduled to assess treatment response after two cycles of rituximab, cyclophosphamide, doxorubicin, vincristine, and prednisolone (R-CHOP) did not show any fluorodeoxyglucose (FDG) uptake in the lymph node areas. However, a comparison between the breast lesion on the initial CT image and on PET-CT images after two cycles of R-CHOP did not reveal any difference in diameter. We observed FDG accumulation in the breast mass with a maximum standardized uptake value of three (Figures 1 and 2). Mammography revealed a circumscribed round-shaped nodule (Figure 3), and the lesion was found to be a well-demarcated hypoechoic mass by ultrasonography (Figure 4). The differential diagnosis included fibroadenoma, breast cancer and lymphoma involving the breast. The lesion was excised and the histopathologic examination revealed that the tumor was composed of spindle-shaped cells with nuclear palisading arranged in interlacing bundles (Figure 5). There were no atypical cells and mitotic figures. A final diagnosis of schwannoma was established by these consistent histopathological findings. The postoperative course was uneventful and the patient was considered in complete response according to PET-CT findings until presenting with central nervous system involvement a month after PET-CT. A regimen of cyclophosphamide, vincristine, doxorubicin, dexamethasone, methotrexate and cytarabine (hyper CVAD) was applied but our patient died at the end of the first year after the diagnosis due to refractory disease. Schwannoma did not recur during the follow-up.

## Discussion

Schwannomas arise rarely within breast parenchyma [6]. In the English language medical literature, 29 previous cases have been identified. According to Guido Bellezza *et al*. most patients were female and the mean age was 47 years. The tumors were most often located in the upper outer quadrant, and tumor size ranged from a few millimeters to 11cm [6]. Our patient presented with a solitary lesion measuring two × one cm in diameter located in the lower inner quadrant. The patient had first noticed the mass 25 years ago. Manifestations of genetic syndromes such as neurofibromatosis types 1 and 2 or schwannomatosis were absent. This benign tumor of the breast may simulate clinically a malignant neoplasm [7]. Taking the long history and the same size of the breast mass in both the initial CT and the interim PET analysis into account, we considered that the breast lesion was a benign one. However, there was still a possibility of residual lymphoma involvement. It is difficult to diagnose intramammary schwannoma on the basis of clinical and radiologic findings [8]. Schwannoma was not diagnosed prior to surgery in our patient and a final diagnosis was established by histopathological examination of the excised mass. The ultrasonographic and mammographic characteristics of the mass in our case were similar to those described in the literature [6,8,9].

It is worth mentioning that we present here the first case of schwannoma of the breast detected on PET, thus far reported. There has been no documentation of the PET findings of intramammary schwannomas. Various schwannomas often have a high level of FDG uptake [10,11]. Schwannomas originate from Schwann cells, which produce the myelin sheath of the peripheral nerves. Schwann cells transport glucose for axonal repolarization. This might be the cause of FDG uptake in schwannomas, although the precise mechanism of FDG accumulation in these benign tumors is not well defined [12].

Schwannomas with high FDG uptake on PET have been described in the brachial plexus [13], liver [14], esophagus [12], posterior mediastinum [15], paravertebral area [11], in the extremities [16], and chest wall [17]. Chang *et al*. reported a case of a schwannoma presenting as an enlarging cervical node demonstrated by PET-CT in a patient with lung cancer that mimicked lung cancer metastasis [18].

## Conclusion

Here we presented a patient with DLBCL who coincidentally happened to have a schwannoma in her breast. PET is a cornerstone procedure in lymphoma management. A PET-positive lesion does not necessarily indicate a diagnosis of lymphoma. False positive PET results are of paramount importance when deciding on the patient’s management. False positives occur in the setting of: inflammation, infection, and necrosis; granulomatous disease including sarcoidosis, thymic hyperplasia, and brown fat or with myeloid growth factor; and rituximab use [19,20]. A diagnostic work-up (a biopsy), just like we did with our patient, should be performed in such patients with lymphoma who have suspected lesions on PET-CT. Schwannoma was a unforeseen diagnosis in the patient described above. PET-positive lesions must be carefully interpreted and rare entities such as schwannomas should be kept in mind in the differential diagnosis. Imaging modalities including PET-CT failed to distinguish breast schwannoma from DLBCL involvement of the breast.

## Consent

Written informed consent was obtained from the patient’s son for publication of this manuscript and accompanying images. A copy of the written consent is available for review by the Editor-in-Chief of this journal.

## Competing interests

The authors declare that they have no competing interests.

## Authors’ contributions

SNE, AEE and SOA analyzed and interpreted the patient’s data regarding the hematological disease. AS was a major contributor in writing the manuscript. MH evaluated PET-CT findings of the patient. All authors read and approved the final manuscript.
